# Concept and Design of a 3D Printed Support to Assist Hand Scanning for the Realization of Customized Orthosis

**DOI:** 10.1155/2017/8171520

**Published:** 2017-11-06

**Authors:** Gabriele Baronio, Paola Volonghi, Alberto Signoroni

**Affiliations:** ^1^Mechanical and Industrial Engineering Department, University of Brescia, Via Branze 38, 25123 Brescia, Italy; ^2^Information Engineering Department, University of Brescia, Via Branze 38, 25123 Brescia, Italy

## Abstract

In the rehabilitation field, the use of additive manufacturing techniques to realize customized orthoses is increasingly widespread. Obtaining a 3D model for the 3D printing phase can be done following different methodologies. We consider the creation of personalized upper limb orthoses, also including fingers, starting from the acquisition of the hand geometry through accurate 3D scanning. However, hand scanning procedure presents differences between healthy subjects and patients affected by pathologies that compromise upper limb functionality. In this work, we present the concept and design of a 3D printed support to assist hand scanning of such patients. The device, realized with FDM additive manufacturing techniques in ABS material, allows palmar acquisitions, and its design and test are motivated by the following needs: (1) immobilizing the hand of patients during the palmar scanning to reduce involuntary movements affecting the scanning quality and (2) keeping hands open and in a correct position, especially to contrast the high degree of hypertonicity of spastic subjects. The resulting device can be used indifferently for the right and the left hand; it is provided in four-dimensional sizes and may be also suitable as a palmar support for the acquisition of the dorsal side of the hand.

## 1. Introduction

In the orthopaedic and rehabilitative fields, there is an increasing interest in the design of customized upper extremity orthosis able to accommodate interindividual anatomy variations of the human body. Prefabricated orthosis and splints can be uncomfortable and unfit, implying various problems such as pain, edema, pressure, and perspiration, often causing important functional loss or even invalidating the therapeutic action of the device [1]. Additive manufacturing technologies, preceded by a reverse engineering process to acquire the geometry and design the orthosis [2], allow the achievement of high levels of customization. For external body parts, although personalized shape modeling can be achieved starting from conventional diagnostic imaging modalities [3, 4], acquisitions by means of 3D scanning devices are often more effective. After being digitized, anatomical data can drive the design of the orthosis through purposeful software. Various rapid prototyping techniques are applicable in the biomedical field, thanks to the continuous evolution of available material and the decrease of the device and production costs [5, 6].

**Figure 1:**
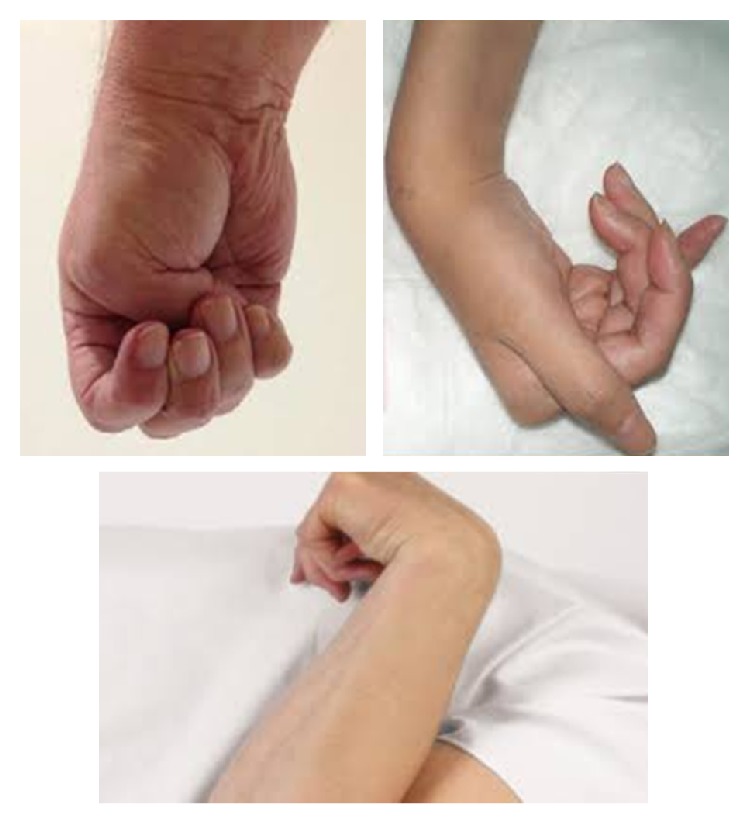
Some examples of spastic hands evidencing hypertonicity.

This work is included in a wider project, named RESHAPER (Reverse Engineering of Self-care and Healthcare Aids for Personalized Empowerment and Rehabilitation), that includes the entire reverse engineering chain for the realization of a customized hand orthosis. In a previous paper, we deeply analyzed and suggested a dedicated 3D scanning procedure for hand acquisition [7]. However, applying it to some classes of patients (e.g., affected by tetraplegia due to spinal lesion or poststroke hemiplegia), the pathological hand scanning could be more laborious and troublesome. Pathologic conditions imply the difficulty to maintain a steady position of the hand and fingers, and, according to the degree of hypertonicity, the patients' fingers tend to close involuntary in a fist (Figure 1). This could cause motion artifacts or lack of acquired areas. Other problems may arise while scanning patients with opposite hypotonicity problems, that is, flaccid hands. Since to realize a personalized orthosis a complete hand geometry is needed (either dorsal or palmar side depending on the kind of device), we devise a strategy to overcome the above limitations by a special support able to maintain the hand in a feasible position so as to avoid lack of data and motion artifacts. Accordingly, the objective of the present work is the design of such a hand support to assist the scanning of patients necessitating a personalized orthotic treatment. The support keeps the hand in a steady position, compatible with the degree of muscular tonicity of the patient and not interfering with the ongoing therapeutic treatment. The hand is positioned for the time necessary to scan the entire geometry of the wrist, hand, and fingers and to obtain a high level of acquired surface accuracy. We realized different versions of the support, modifying gradually its design, in accordance with technical and clinical specifications, and the optimal positioning of the fastening devices, always respecting patient comfort.

## 2. Related Work

For hand orthosis construction, two paths are typically followed, which however evidence major limitations. In the first one, a complete contact procedure is adopted to create a hand-made personalized orthosis in low-temperature thermoplastic material (LTT). The typical workflow followed by orthopaedic technicians [8] starts with pattern creation, drawing around the patient's wrist and hand on a piece of paper, making attention to relevant landmarks as pressure points. The pattern is refined, and the sheet of paper is cut along the drawing and tested on the patient; later, the material (typically LTT), the padding material, and the fasteners are chosen. The cut paper is transferred on the LTT, that is, later cut and fitted to the patient and adjusted; fasteners are fixed to the orthosis, and finally, optional adjustments are made as the creation of ventilation holes. This type of fabrication is regularly employed in a clinical environment, even if it can be very laborious, time-consuming, and quite painful for the patient.

The second approach includes the use of a 3D scanner to acquire a plaster cast manufactured directly on the hand. A high-metric precision is achieved, and problems related to involuntary patient movements are supposedly removed. However, it is evident how this procedure is time- and material-consuming, invasive, and can be uncomfortable for the patient. Studies that follow this approach do not usually consider the acquisitions of subject affected by spastic or other invalidating pathologies [9]. Possible ways to overcome the inability to scan internal and intricate surfaces due to line-of-sight limitations, occlusions, and anatomical complexity (the hand is a geometrically complex district) are again invasive and difficult to apply on patients without producing discomfort and fatigue [10].

An improvement in terms of comfort, time, and material saving can be the direct scanning of the anatomy of interest [11] to obtain a model that can be integrated in a reverse engineering approach that ends with the 3D printing of the customized orthosis. In literature, such method is typically applied for anatomical portions which are easy to acquire because of the absence of occlusion issues (e.g., leg, arm, forearm, and face, excluding hands and fingers) [9, 12]. High levels of customization can be achieved by following a reverse engineering approach, usually consisting of three main stages [13, 14], which are critically analyzed in [2]: (1) scanning of anatomical parts, (2) processing of the acquired geometry using CAD software, and (3) creation of the device using additive manufacturing technologies. In literature, there are a few studies which analyze the whole hand orthosis realization process [15, 16]; others concentrate on specific stages, taking anatomy acquisition as provided by suitable systems [17–19]. In [7], we tested, on healthy subjects, different scanning methodologies and partially solved the problem of occlusions and involuntary motions by a deformable multiview alignment solution. However, to our knowledge, no studies tried to overcome the problem of lack of acquired data due to spasticity attitude. We address this issue by the design and use of a special support to keep the hand open and as close as possible to the desired position.

## 3. Materials and Methods

The design of the hand acquisition support was conceived as an iterative procedure, attributable to *planning and task clarification*, *conceptual, and embodiment design* and *detail design* [20].

### 3.1. Planning and Task Clarification

To our knowledge, no studies dealt with the design and realization of support devices useful to assist the 3D scanning of the hand anatomy, probably due to the scarcity of acquisition procedures of the hand including fingers that are very complex to acquire, especially in cases of patients affected by neurologic disorders.

To better understand the problem of immobilization and positioning of a patient's hand during the acquisition, two representative examples are presented among those analyzed at the rehabilitation clinic Domus Salutis (Brescia, Italy). Hand acquisitions were performed using the structured-light scanners *Cronos 3D Dual* and *Insight* by *Open Technologies Srl*, (Rezzato, Italy), http://www.scanner3d.it/en/. The scanning procedure identified in [7] was applied here on subjects needing an auxiliary device in order to keep their hands in the proper position during scanning. The tendency to close the hand in a fist was confirmed by the preliminary tests, and in some cases, it produced useless acquisition due to the lack of internal areas, making the use of an auxiliary device to keep the hand open necessary. Moreover, for some patients, beyond the expected issues in maintaining a steady position, we observed additional difficulties in assuming the voluntary supine position of the hand, which is instead useful to facilitate the palmar side acquisition.

**Figure 2:**
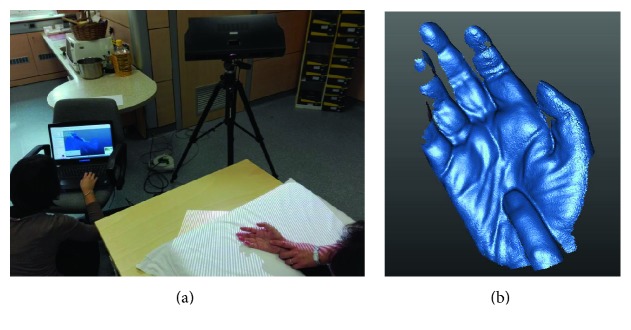
Hypotonic patient: (a) 3D scanning and (b) acquired data.

In the first case, shown in Figure 2(a), the low tonicity of the patient's hand allows the limb to be placed in an almost optimal position for the acquisition of the hand side, with a minimum intervention of the therapist (visible finger index). The acquisition result (Figure 2(b)) highlights two typical issues: the surface of the fingers is not sufficiently complete (more views needed), and the thumb moved during the scanning.

**Figure 3:**
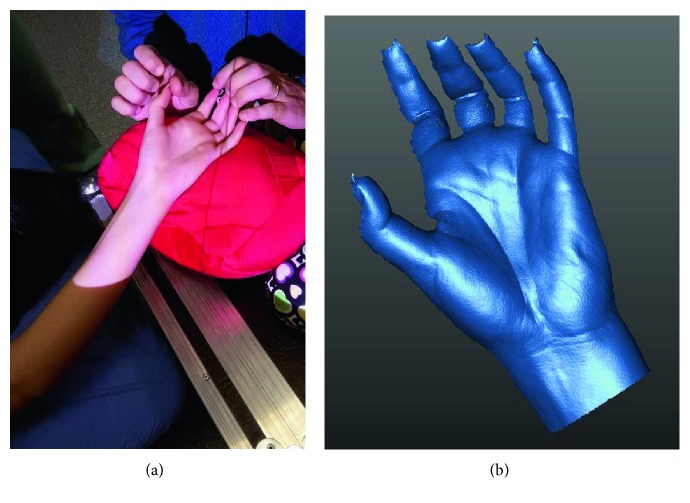
Hypertonic patient: (a) 3D scanning and (b) acquired data.

In the second example, shown in Figure 3(a), the therapist's intervention in the acquisition phase is greater, and the patient's hand must be held firmly using the corresponding fingers of the therapist. However, the 3D acquisition result is more satisfactory for both completeness and accuracy (Figure 3(b)).

Based on the considerations emerged among clinicians, therapists, and engineers, by the qualitative analysis of the results of the above and other scanning tests, the need to improve the scanning process of the palmar side during hand acquisition clearly emerged. Here, we address the emerged issues by the design and the 3D printing fabrication of a dedicated immobilization support according to the following requirements list:

- Bilateral (right and left hand) use
- Bifrontal (palmar and dorsal) acquisitions
- Low cost
- Easy to position and fix
- Acquisition of the anatomy in a physiologic position
- Wearable by different hand sizes

### 3.2. Conceptual and Embodiment Design

Since the scope of the present work was to satisfy a completely new necessity, that is, to acquire hand geometry including fingers using 3D scanners in subjects with compromised functionality of their limb, the design phase paid attention to device functionality and the possibility to easily and quickly obtain prototypes with different geometry and increasing complexity.

A satisfactory solution was obtained here through iterative process that consist in identifying additional technical specifications, assessing progressive geometry modifications thanks to 3D printing. With the future works, we will optimize the device, performing functionality tests and finite element analysis.

**Figure 4:**
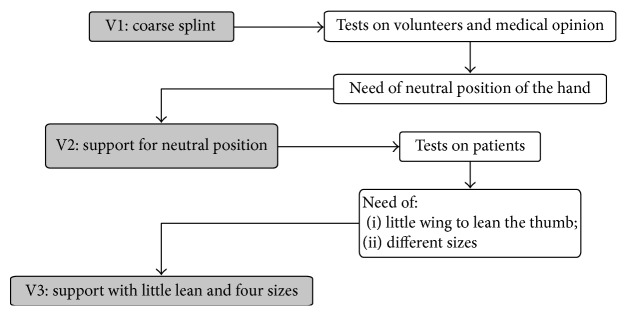
Operational workflow for the support design and realization.

The operational workflow is reported in Figure 4.

*Version 1* (*V1*) was realized to satisfy initial technical requirements:

- Shape compatible with human hands that could be applied on both left and right hand
- Support made with additive manufacturing (3D printing)
- Arrangement of eyelets to fix the support to the hands of the patients.

*Version 2* (*V2*) included additional specification:

- The support should be designed with a shape reproducing the neutral position of the hand.
- The eyelets should be more numerous, to allow a flexible fixing of the fingers.

The neutral posture of the hand is a position of equilibrium without active muscle contraction, and it is considered by the therapists as the optimal position to treat spastic hand. Heo et al. [21] described such position as follows: the wrist is extended 20° in neutral radial/ulnar deviation, the metacarpophalangeal (MCP) joints are flexed approximately 45°, the proximal interphalangeal (PIP) joints are flexed between 30° and 45°, and the distal interphalangeal (DIP) joints are flexed between 10° and 20°.

*Version 3* (*V3*) was realized to fulfill more specific requirements:

- The thumb had to be kept in opposition to other fingers.
- Different sizes of the support must be realized.
- Velcro fastening chosen for V2 should be replaced with another fastening solution.

**Figure 5:**
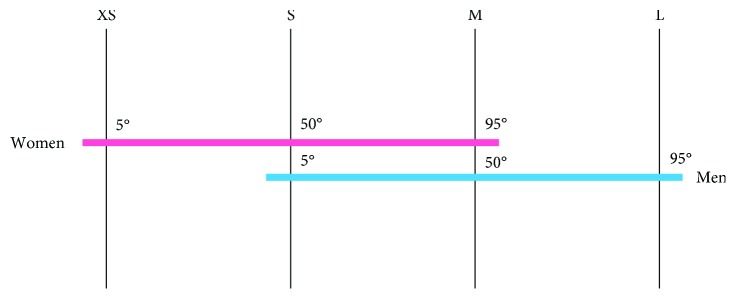
Sizes.

Anthropometric measurements were considered to realize different sizes of the support. Pheasant [22] described measures of the hands distinguishing men and women, reporting the 5th, 50th, and 95th percentiles. Since in literature a universal definition of hand sizes is not present, we decided to combine in a suitable way the percentiles and we defined four different sizes: size XS (5th percentile women), size S (50th percentile women and 5th percentile men), size M (95th percentile women and 50th percentile men), and size L (95th percentile men) (Figure 5).

**Figure 6:**
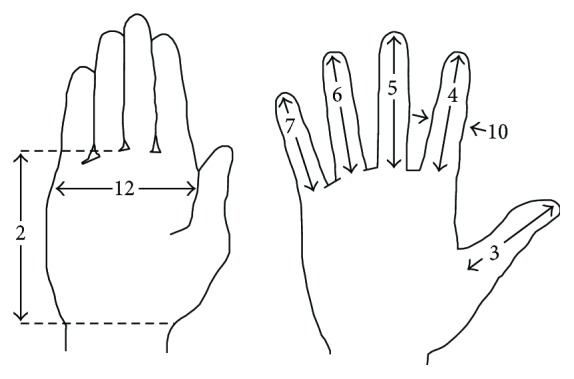
Reference measurements (adapted from pheasant [22]).

The measures that were considered in this work are illustrated in Figure 6: palm length (2), thumb length (3), index finger length (4), middle finger length (5), ring finger length (6), little finger length (7), index fingers breadth (10), and handbreadth (12) [22]. The total length of the fingers was not enough, due to the different angular position of the three phalanges. The length of each phalanx of each finger was reconstructed through relations found by Buryanov and Kotiuk [23] that measured the relative distances (in percentage) to the joints of the fingers. Formulas we used were as follows:

(1) $$\begin{array}{c} P3=D\cdot fl,\\ P2=D\cdot fl,\\ P1^{*}=D\cdot fl, \end{array}$$

where (i) P3 is length of the third phalanx; (ii) P2 is length of the second phalanx; (iii) P1^∗^ is the web height (to the metacarpophalangeal joint); (iv) fl is external length of the finger (from the tip to the thenar webbing); (v) *D* is tabulated value in percentage from [23].

The length of the first phalanx P1 was deduced from the difference between the total length of the finger (fl) and the length of the second and third phalanges (P2 and P3), adding the web height to arrive up to the MCP joints. We considered for each phalanx the biggest value of the four fingers.

### 3.3. Detail Design

Supports have been modeled with CAD software and realized by 3D printing using a FDM printer (Stratasys “Dimension BST 1200es”) employing ABS material (ABSplus, Tensile Strength: 37 mpa) with a layer thickness of 0.254 mm and build size 254 × 254 × 305 mm.

The thickness of the support was equal to 3.5 mm [16]; later, it was increased to 5 mm to improve the stiffness of the device.

This printer is suitable for prototype design and testing, ensuring sufficient stiffness characteristics to the product. For larger-scale usage, further optimizations and fabrication with professional 3D printers (with the use of more advanced and certified medical materials) are to be taken into consideration.

## 4. Results and Discussion

**Figure 7:**
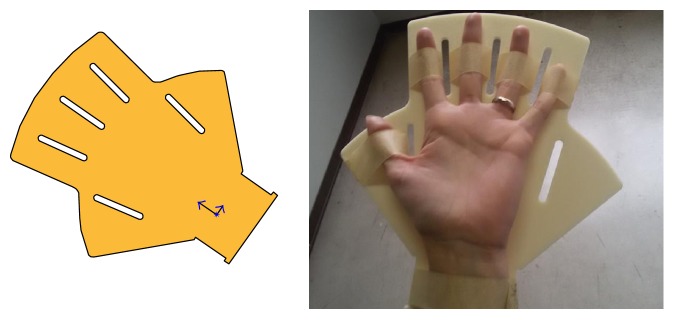
Support version V1: design, fabrication, and use.

*V1* was tested on a volunteer hand, and it was fixed using masking tape (Figure 7). The palm-like shape was considered appropriate, but some limits emerged: the flat shape could be uncomfortable (even for healthy subjects) if not even painful (especially for patients) and the completely open position of the hand (0° flexion of fingers and wrist) is not to be considered of therapeutic value, rather it could be detrimental for the treatment.

**Figure 8:**
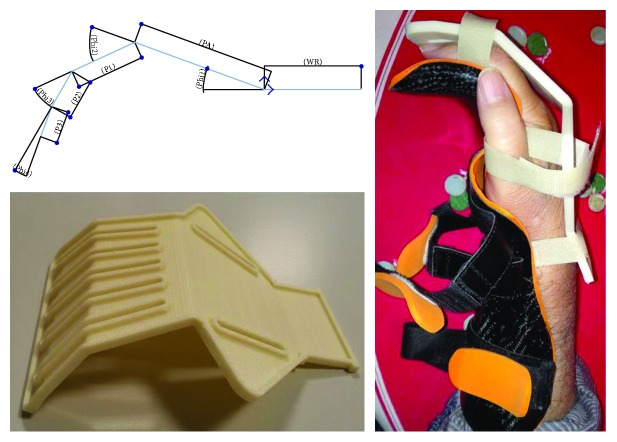
Support version V2: design, fabrication, and use.

*V2* had 5 eyelets instead of 3 to fix the fingers excluding the thumb. The design and a prototype are reported in Figure 8, while in Table 1, the angular measurements, chosen in accordance with clinicians and occupational therapists, are reported. The principal limitation of this version was the lengths of the different hand portions that was chosen based on a volunteer's hand dimensions. Since the masking tape used in *V1* resulted discomfort, it was replaced by Velcro strips.

**Figure 9:**
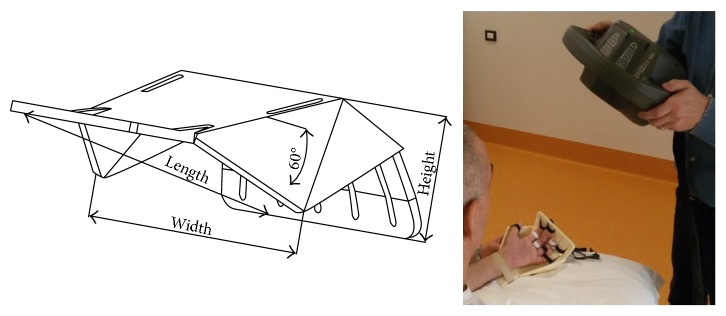
Support version V3: design, fabrication, and use.

In *V3*, little wings were realized in correspondence of the thumb, to facilitate its opposition to the other fingers. They were bent with an angle of 60° with respect to the hand plane (Figure 9).

The established lengths in this work are reported in Table 2, where PA is the palm length (pheasant measure [22], number 2 in Figure 4, reduced by the web height P1^∗^); P1, P2, and P3 are the phalange length; WB is the wrist breadth that we considered equivalent to the handbreadth (number 12 in Figure 4); WR is the wrist length that we established as fixed for each size; length, width, and height are the overall dimensions of the support (Figure 9).

Fixing was performed replacing Velcro with synthetic leather closed with buckles since, in our context, Velcro strips are not very practical and may cause skin irritation.

Fulfillment of the support requirement list is compared in Table 3. The analysis proposed in Table 3 shows a comparison, where for each requirement, “-” was assigned if the requirement is not satisfied, “+” if the requirement is satisfied, and “++” if the requirement is well satisfied.

It is noteworthy to observe that the shape of the support *V3* is also suitable for a bilateral usage, that is, as a support for the hand palm for correct scanning in case of acquisition of the dorsal side of both right and left hands, for possible design and realization of dorsal orthoses.

It is also important to clarify that reproducibility of the hand positioning on the support was not a cogent requirement, since the orthosis can be finely corrected via suitable software tools. Moreover, intersubject reproducibility is difficult to define due to the large differences in the degrees of pathological conditions (e.g., more or less hypertonicity).

The design methodology used in this experimental work was highly iterative/interactive and characterized by a continuous comparison between clinical and design requirements; by testing the first version of the support, we understood where to improve it; the second version was obtained first and then the third one. In this context, the use of additive manufacturing techniques for the fabrication of various support prototypes has proved to be fundamental. In a suitable time, we succeeded in obtaining very different morphology support samples without having to resort to more expensive and time-consuming manufacturing processes.

## 5. Conclusion

A supporting device was developed based on the hand immobilization requirements when acquiring the palmar hand side of mobility-limited patients by means of 3D scanners.

The iterative designing process and the interaction between clinicians and designers made it necessary to design and produce more versions of the supporting device.

The use of additive manufacturing techniques in the development of the device allowed us to follow and pursue the design process successfully.

The resulting product, besides being usable indifferently on the right or left hand, may be suitable as a palmar support in the acquisition of the dorsal side of the hand.

As a next step in the project, we will extend the usage of the designed supporting device to a wider sample of patients and acquired data will be used for the design of custom orthoses.

## Figures and Tables

**Table 1:** Angular measures of support for neutral position of the hand (for parameter significance, see Figure 8).

Angle name	Degree
phi1	20°
phi2	45°
phi3	35°
phi4	10°

**Table 2:** Considered values (in mm) for each size.

	Size S	Size XS	Size M	Size L
PA	78	86	93	102
P1	39	43	47	50
P2	23	25	28	30
P3	18	20	22	24
WB	69	77	85	95
WR	60	60	60	60
Length	194	207	219	232
Width	175	177	179	181
Height	60	65	71	76

**Table 3:** Support version comparison.

Requirement list	Version V1	Version V2	Version V3
Right and left hand use	+	+	+
Palmar acquisitions	+	++	++
Low cost (3D printing time)	++	+	+
Easy to position and fix	−	+	++
Acquisition of the anatomy in a physiologic position	−	+	++
Wearable by different hand sizes	−	−	++
